# Shared decision making by United Kingdom osteopathic students: an observational study using the OPTION-12 instrument

**DOI:** 10.1186/s12998-019-0260-0

**Published:** 2019-09-05

**Authors:** Dévan Rajendran, Jane Beazley, Philip Bright

**Affiliations:** Research Department, European School of Osteopathy, Boxley House, The Street, Boxley, Kent, ME14 3DZ United Kingdom

**Keywords:** Shared decision making, Patient centered care, OPTION-12 instrument, Osteopathy, Education, Clinical teaching

## Abstract

**Background:**

At the crux of patient centred care is Shared Decision Making (SDM), which benefits patient and practitioner. Despite external pressures, studies indicate that SDM remains poorly practised across a variety of healthcare professions. The degree of SDM engagement within United Kingdom osteopathic undergraduate teaching clinics is currently unknown.

**Methods:**

In 2014 we used the reliable and validated OPTION-12 (O12) instrument to calculate a score that reflected the degree of SDM utility in one United Kingdom Osteopathic Educational Institute’s teaching clinic. We also aimed to compare these scores with those previously obtained for physiotherapists working within the United Kingdom’s National Health Service. Student-patient initial and follow-up encounters were audio recorded, transcribed and scored using the O12. Comparisons between the following O12 scores were performed: the Osteopathic Educational Institute’s 4th and 3rd year students; the Osteopathic Educational Institute’s student’s initial and follow-up patient encounters; the Osteopathic Educational Institute’s students and National Health Service physiotherapists.

**Results:**

We analysed 35.5 h of transcribed data from 30 student-patient encounters (7 initial: 23 follow-up). An O12 score of 0.6% (range 0–10.4%) was calculated. No significant differences were found between year groups or encounter types. Significant differences were found compared to National Health Service physiotherapist (score = 24.4%): (U = 144, z = 4.25, *p* < 0.0005); although both scores are below the 60% threshold for competent SDM behaviour.

**Conclusions:**

Undergraduate osteopaths did not appear to engage in competent SDM behaviours, implying traditional and paternalistic styles of decision making that align with results from other manual therapy professions. Students in this study did not practise competent SDM behaviours. Effective educational strategies are required to ensure SDM behaviours reach competent levels.

## Background

Shared Decision Making (SDM) has been defined as “…an approach where clinicians and patients make decisions together using the best available evidence” [1]. An ethical imperative is found at the heart of patient centred care, a paradigm in which clinicians and patients work together using best available evidence to agree a fully informed plan for that patient’s treatment or care [2]. The process requires equal collaboration of both parties, with patients actively encouraged to deliberate on information presented to them and to communicate their personal preferences to the clinician [3]. A good therapeutic relationship and a supportive environment are pre-requisites for patients to: understand information presented to them; explore what is important to them; deliberate on all available options and express themselves openly [4].

Patients’ desires to be involved in decision making were first identified in the mid-1970s [5], but the concept of SDM was only articulated in 1999 [6]. Since then, SDM has been the subject of ongoing discussion, research and policy initiatives across the developed world [7].

There are many reported benefits of using SDM as a clinical tool; SDM adept clinicians are able to collaborate with their patients and note improvements in: health outcomes and reduced referrals for clinical testing [8]; improved patient satisfaction and adherence to treatment [9] as well as increased patient autonomy and engagement [10]. A number of negative practitioner beliefs/perceptions have also been identified: SDM takes too much time; patients do not want to be involved in decision making [4] and giving patients power to decide is an abdication of professional duty [2]. These beliefs may partly be related to when practitioners were trained: those qualifying before SDM was introduced are more likely to adopt a ‘doctor knows best’ approach to patients [11].

Paradoxically though, despite professional pressures on clinicians to use SDM, patients may not actually be interested in evidence based medicine; anecdotes appear to trump scientific evidence [12]. Ironically, patients may appear quite resistant to engaging with SDM and Towle et al. [13] identified a number of barriers including: satisfaction with the existing relationship leading to a low motivation for change; perceptions that change is outside of a patient’s control; change will risk any existing rapport; a lack of skills required to change communication patterns. Additionally, patients often feel vulnerable given that clinicians are generally more knowledgeable, or are perceived as such, and thus hold power in the relationship [14]. Rectifying this power imbalance requires greater patient knowledge and is reliant on clinicians preparedness to give patients the opportunity to be involved [15].

Poor general health of a patient and cognitive impairment such as dementia have been identified as non-modifiable factors that impact on SDM engagement [15]. Poorly modifiable patient factors include low health literacy, low numeracy or patients who come from culture backgrounds that discourage autonomous decision making [4]. In some cases, decision making may include opinions and beliefs of family and/or friends, adding additional layers of complexity to engagement with SDM [14]. Finally the impact of media, social and otherwise, may also impact on patient’s willingness to engage with SDM [16].

Within the United Kingdom, the National Institute for Health and Care Excellence has adopted SDM as a quality standard, stating patients must be “actively involved in shared decision making and supported by healthcare professionals to make fully informed choices about investigations, treatment and care that reflects what is important to them” [17]. The components of SDM include: 1) Define the problem to be addressed; 2) Present the options; 3) Patient and clinician discuss pros and cons of each option to include; 3a) Clinicians knowledge about risks, benefits, costs, convenience; 3b) Patients ability or self-efficacy in following through with tests, medications, procedures, required behavioural changes, referrals [18]. It is also important to recognise that SDM is a process and Makoul et al. (2006) go on to state, ‘it is essential that physicians and patients arrange follow-up to track the outcome of decisions that have been made or reach resolution on those that have not’.

The United Kingdom’s National Health Service provides healthcare to the populace and estimates suggest that over a million patients are seen by the National Health Service every 36 h [19]. In 2009, the National Health Service published their constitution, part of which committed to provide patients with sufficient information for them to be able to participate in discussions and decisions regarding their care [1], which embeds SDM into the National Health Service via the ‘Right Care Shared Decision Making Programme’ [20]. Similarly, the General Medical Council, the statutory regulatory body for United Kingdom medical doctors, obliges doctors to work in partnership with patients by listening to their concerns and preferences, providing them with the information they need, in a way they can understand, in order to make a joint decision about their treatment or care [21]. Although the current Standard of Proficiency for United Kingdom physiotherapists published by the Health and Care Professions Council, contains no explicit requirement for SDM [22], physiotherapists working within a National Health Service setting would be obliged to commit to the National Health Service’s ‘Right Care Shared Decision Making Programme’.

Self-determined United Kingdom patients do selectively seek complementary care outside of the National Health Service, with osteopaths being one of the regulated healthcare professionals who are sought out; some of whom are contracted to work within the National Health Service [23]. The 5000 United Kingdom osteopaths carry out approximately seven million encounters a year and are statutory regulated by the General Osteopathic Council [24]. The General Osteopathic Council are responsible for setting the professional standards and codes of practice for the United Kingdom osteopathic profession. The current Osteopathic Practice Standards came into force in September 2012 and is nearly identical to General Medical Council’s guidance on good medical practice. Within the Osteopathic Practice Standards, the concept of SDM is explicitly referred to; standard A5 states that osteopaths must “work in partnership with patients to find the best treatment for them”; guidance note 1 states, “You should encourage patients to ask questions about their treatment and to take an active part in the treatment plan and any decisions that need to be made [24]”. This obligation remains within draft revised guidelines (relisted as A3.2) that are scheduled for publication in September 2019.

As a statutory regulator, the General Osteopathic Council is responsible for handling patient complaints about osteopaths. Complaints are screened, investigated and if certain threshold criteria are reached, ultimately heard by a panel of the Professional Conduct Committee. In 2014 incidence rate of concerns raised were < 0.004% and the number of serious cases heard by the Professional Conduct Committee panel involved < 1% of United Kingdom registered osteopaths [25]. The largest component of patient concerns raised are about professional conduct, which includes ‘no shared decision-making with the patient’ [26] Despite the most recent Figs. (2017) showing a five year low in the number of patient complaints and concerns against United Kingdom osteopaths, failures in professional conduct including shared decision making remains the largest component [27].

The General Osteopathic Council is also responsible for validating Osteopathic Educational Institutes’ training of undergraduate students, ensuring they reach the prerequisite level of competence set out within the Osteopathic Practice Standards, to be able to join the register. Clinician training traditionally focuses on embedding problem-solving communication skills, which are different from those required for SDM behaviours. Problem solving training enables novice clinicians to focus on diagnosis formulation and patient management planning, but once acquired these forms of communication skills may become habitual and difficult to change in the future [26]. Whist embedding explicit SDM training into a clinician’s early medical education should ensure that appropriate SDM behaviours become the norm for these future medics, there is some evidence that even if medical students are taught SDM at undergraduate level, once they become mentored by mature clinicians, they may adopt that clinician’s style of communications, including any ‘bad habits’ (non SDM behaviours) [27].

There is some preliminary evidence suggesting a relationship exists between the exit degree level of undergraduate osteopathic education, a practitioner’s conception of practice and an osteopaths’ engagement with SDM behaviours: the latter being categorised as either ‘clinician-led’, ‘patient-led’ or ‘shared’ [28]. Although SDM behaviours have been assessed across a range of other health professional settings and across a number of countries [29], the extent to which United Kingdom osteopathic students use SDM within clinical encounters is currently unknown. At the time that this study was conducted, SDM was not an explicit topic within the syllabus of the Osteopathic Educational Institute, although the concepts and practicalities of enhancing patient communication and obtaining consent had been taught to the clinical students taking part in this study.

Nonetheless, in their systematic review, Couët et al. noted that those studies which included an intervention designed to improve qualified clinicians’ SDM behaviours, all showed positive improvements in post-intervention O12 scores; ‘interventions’ included the introduction of decision aids as well as training [20].

Additional external pressures on adoption of SDM behaviours have been identified and include commercial, regulatory and legal pressures on practitioners. There is a shift toward viewing the patient as a ‘consumer’ and an acknowledgement that patients are now often ‘informed’ about their problems even prior to consultation with a practitioner [26]. A 2014 United Kingdom Supreme Court found a case of medical negligence proven and in doing so reviewed the duty of disclosure a doctor has towards a patient. Known as the Montgomery Precedent, it outlines the rights of patients to be told of ‘material risks’ inherent in a treatment in order for the patient to be able to make an informed decision about whether they wish to incur those risks [28]. Materiality was defined as “whether a reasonable person in the patient’s position would be likely to attach significance to the risk, or the doctor is or should reasonably be aware that the particular patient would be likely to attach significance to it.” [29].

In 2014, Jones et al published a study that used a validated instrument to determine the degree to which 12 National Health Service physiotherapists (including ‘newly qualified’ or Band 5 practitioners) working in a hospital setting, used SDM behaviours [30]. The publication included score data obtained from the instrument that was detailed enough to allow any future studies to be able to make a comparison with their data. We therefore aimed to replicate the study in so far as we used the same tool and method of capture. This allowed us to both determine the degree to which clinical students within one United Kingdom Osteopathic Educational Institute’s teaching clinic used SDM and also statistically compare these to Jones et al.*’s* data.

## Methods

Ethics approval was obtained from the Osteopathic Educational Institute’s research ethics committee and we captured SDM behaviour using the OPTION-12 (Observing Patient Involvement) scale, a previously validated instrument [31]. This reliable instrument measures the prevalence of practitioner SDM competencies captured within the verbal communications that occur during a patient-practitioner interaction. The OPTION-12 (O12) consists of twelve SDM behaviours, each ranked on a five point scale (0 to 4; 0 = not observed; 4 observed and executed to a high scale). Higher scores indicate greater competency in the observed SDM behaviour; the highest possible summed score for all twelve behaviours is 48 points. Each score is converted to a scaled percentage; a total O12 score of 60% deemed the lowest meaningful competence of SDM [31].

### Setting

Data collection took place in the out-patients teaching clinic of one Osteopathic Educational Institute over a seven-week period from June to August 2014.

#### Participants & recruitment

All clinical students (third and fourth year) were invited via email and we used convenience sampling for patient selection. To reduce the possibility of participants modifying their usual SDM behaviour, all students and patients were informed that this was a generic observational study utilising audio recordings to capture practitioner-patient interactions.

#### Data collection and analysis

We obtained written consent from all students, patient and clinical tutors prior to enrolling them in this study. Verbal interaction that took place during the student-patient encounter was captured using two Olympus DM-5 digital audio recorders; the investigator remained outside of the treatment room during all recordings. The recordings were transcribed by JB using Microsoft Word (Microsoft Corporation, Redmond, WA, USA), Express Scribe (NCH Software Inc., Greenwood Village CO, USA) and then anonymised. To ensure consistency, all transcriptions were analysed using the O12 by JB, who had undergone training to use this instrument. To ensure reliability, a second independent O12-trained coder analysed 10% of the transcribed data and an inter-rater reliability of score was calculated using Gwet’s first-order Agreement Coefficient [32], which obviates the kappa paradox [33]; if ambiguities were identified, these were discussed and if consensus not reached, a third coder was consulted.

O12 scores were tabulated using Microsoft Excel v14 (Microsoft Corporation, Redmond, WA, USA), summated and scaled to produce percentage scores; each O12 point equated to 2.03 percentage points. Wilcoxon’s Signed Rank test was used to determine statistically significant differences between the following O12 scores; Osteopathic Educational Institute 4th and 3rd year students; the student’s initial and follow-up patients; the students and National Health Service physiotherapists [30]. All calculations were performed using IBM SPSS Statistics for Windows, Version 25.0. (Armonk, NY: IBM Corp).

## Results

We audio recorded treatment sessions from thirty two student-patient dyads: demographic data for the dyads are presented in Table 1. Twenty three sessions were follow-up treatment encounters, each lasting 40 min and nine from initial patient encounters lasting 80 min. The quality of audio recordings from two interactions were unusable, both were from initial encounters. Thus our results are based upon anonymised and transcribed data from over 35 h of recordings derived from 30 student-patient interactions (7 initial encounters and 23 follow-up encounters). Agreement Coefficient 1 inter-rater reliability score was 0.74, equating to a reliability of > 70%; one transcript required discussion and score consensus obtained, the third assessor was not required. A tally of the number of students displaying each of the O12 SDM behaviours is found in Table 2.

**Table 1:**
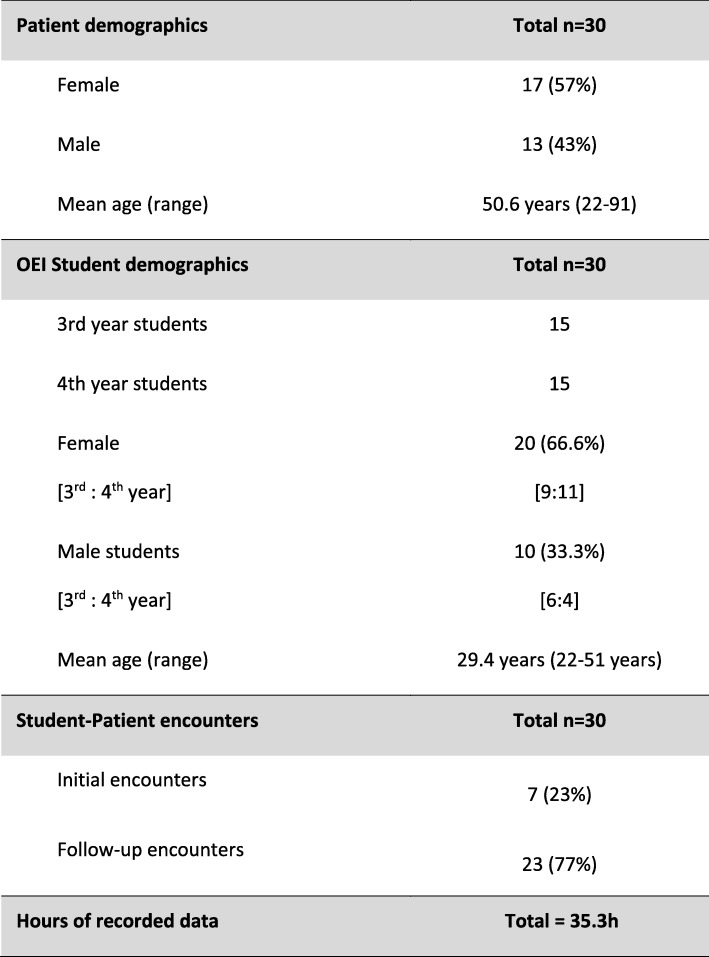
Participant demographic and background data

**Table 2:** O12 - number of items identified in initial and follow-up encounters of OEI students

OPTIONS items	Initial encounters (*n* = 7)	Follow-up encounter (*n* = 23)
1: The clinician draws attention to an identified problem as one that requires a decision making process	0	2
2: The clinician states that there is more than one way to deal with the identified problem	0	1
3: The clinician assesses patient’s preferred approach to receiving information to assist decision making	0	0
4: The clinician lists ‘options’, which can include the choice of ‘no action’	0	0
5: The clinician explains the pros and cons of options to the patient (taking no action is an option)	1	1
6: The clinician explores the patient’s expectations (or ideas) about how the problem(s) are to be managed	0	3
7: The clinician explores the patient’s concerns (fears) about how problem(s) are to be managed	0	0
8: The clinician checks that the patient has understood the information	1	0
9: The clinician offers the patient explicit opportunities to ask questions during decision making process	0	0
10: The clinician elicits the patient’s preferred level of involvement in decision making	0	0
11: The clinician indicates the need for a decision making (or deferring) stage	0	0
12: The clinician indicates the need to review the decision	0	0
Total number of OPTION-12 items observed (number of OEI students)	2 (*n* = 2)	7 (*n* = 3)

### Initial encounters

Out of the seven initial encounters, two (28.5%) contained evidence of a student performing one different SDM behaviour each. Each student achieved a 1 point score (i.e. ‘Brief or perfunctory attempt’) for their O12 item. One student displayed O12 item 5 behaviour and the other displayed item 8 behaviour. In the remaining five initial encounters, none of the students were observed exhibiting any of the SDM O12 behaviours. The modal and median point scores for each of the 12 behaviours were 0, ‘not observed’ (Table 3). Students achieved an O12 score of 0.6% for the initial encounters (Table 4).

**Table 3:**
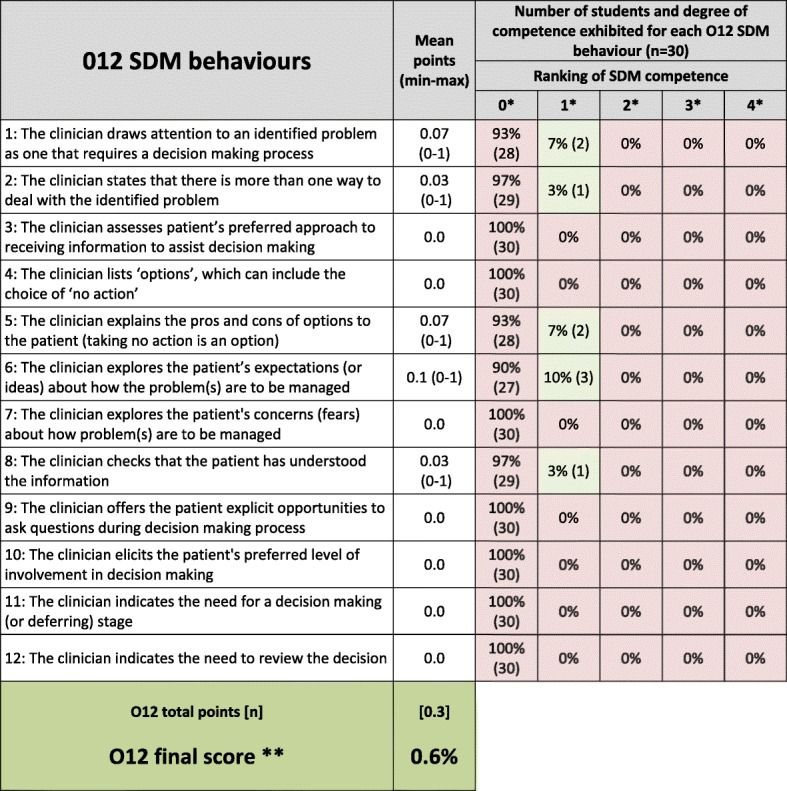
Accumulated distribution of 012 scores for both types of student-patient encounters

*O12 competence point allocation: 0 = No attempt; 1 = Brief or perfunctory attempt; 2 = Baseline skill level; 3 = Behaviour exhibited to a good standard; 4 = Skill exhibited to a high standard

**60% is baseline for minimally accepted level of SDM competence (Elwyn et al. 2012)

**Table 4:**
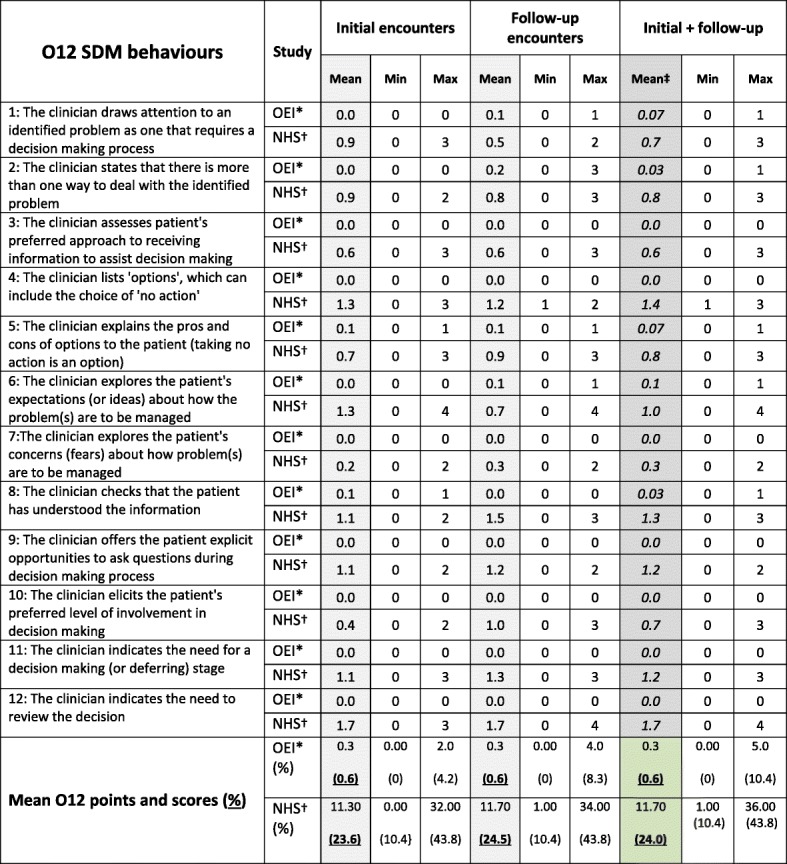
Comparison of NHS and OEI O12 Point Scores

Key:  Final O12 points obtained and converted to (%) scores

* OEI data are replicated from Table 2

†Original Jones et al. (2014) data are reproduced for ease of comparison

‡These data were used to test for statistical differences between NHS physiotherapists and the OEI students

### Follow-up encounters

Of the twenty three follow-up encounters, a total of three students (13%) were identified as performing four separate SDM behaviours (13%) and all of these behaviours were scored as 1. Three students displayed two behaviours each and one displayed three. All three students achieved a 1 point score for item 6; two achieved a 1 point score for items 1 and 5, and two achieved a 1 point score for items 2 and 8; no other SDM behaviours were observed. Students achieved an O12 score of 0.6% for the follow-up encounters (Table 4).

### Combined scores from both types of encounters

Taken together, a total of five students (17%) were observed exhibiting SDM behaviours; two (7%) exhibited one SDM behaviours each, two (7%) exhibited two SDM behaviours each and one student (3%) exhibited three SDM behaviours. The highest score achieved on any of the observed behaviours was 1*.* Across all patient encounters the students scored 0.3 points (range = 0 to 1), which equates to an O12 score of 0.6% that ranged from 0 to 10.4% (Table 4).

No student was observed demonstrating all twelve items on the O12 score and no student was observed performing the following seven O12 items: 3); 4); 7); 9); 10); 11); or 12). The most commonly observed behaviour was for item 6, which was seen in 3 (7%) students. Two students (7%) were observed demonstrating items 1 and 5 and another two students (7%) were observed demonstrating a single behaviour each: items 2 and 8.

Visual inspection of O12 score distributions between the year groups revealed them to be similar. O12 scores for third year (mean rank 16.0) and fourth year students (mean rank 15.0) were not statistically different, U = 105.50, z = − 0.445, *p* = 0.775. The distribution of O12 scores between the types of patient encounters was not similar on visual inspection. O12 scores for initial encounters (mean rank = 15.1) and follow-up encounters (mean rank = 16.86) were not statistically different; U = 90.00, z = 0.718, *p* = 0.667.

### Comparison to National Health Service physiotherapists

We abstracted and tabulated the summed and compared data from Jones et al. (2014) with the results obtained from the Osteopathic Educational Institute students (Table 4). Visual inspection of O12 score distributions between the physiotherapy and osteopathy groups revealed them to be dissimilar. O12 scores for Osteopathic Educational Institute (mean rank 6.5) and National Health Service (mean rank 18.5) were statistically different, U = 144, z = 4.25, *p* < 0.0005.

## Discussion

We audio-recorded, transcribed and scored 30 third and fourth year clinical student practitioner-patient interactions using the O12 instrument to assess evidence of SDM behaviours with initial and follow-up patient encounters. Elwyn et al. (2005) recommended that a total O12 score of 60% should be considered the threshold for meaningful competence of SDM [31]. At the OEI, students achieved a mean O12 score of 0.6% (initial encounters 0.6%, follow-up encounters 0.6%). Across both encounters, seven of the twelve O12 behaviours were not displayed by any students.

Within the teaching clinic, initial patient encounters are twice the length of a follow-up encounter (80 mins vs 40 mins respectively). This extra time is designed to give students the time to examine, diagnose and then to explain their examination findings, diagnosis and proposed management plan to their patients. Nonetheless although there appeared to be a trend towards more students displaying SDM behaviours within the initial encounter (2 of 7 students) compared with 3 of 23 students in the follow-up group, the O12 scores were not statistically different between the encounters.

Similarly, we found no significant differences between O12 score of the third and fourth year students, which implies that the extra year of clinical teaching and supervision does not result in a higher engagement of SDM within the undergraduate teaching clinic. Whilst this appears to contradict findings by Thompson et al. (2014), this might reflect the technical focus of this Osteopathic Educational Institute’s curriculum, which was in operation at the time of this study.

### Comparison with other manual therapists

Significant differences were found between the scores from our study (mean = 1.5%) and those obtained by Jones’ et al. [30] National Health Service physiotherapists (mea*n* = 24%). However all the physiotherapists were graduated and had varying degrees of experience: three were Band 5 (‘newly graduated’) who might be comparable to the year 4 students in terms of numbers of years since commencing studies, five were Band 6 (‘experienced or specialist’ grade) and four were Band 7 (‘advanced practitioner’ grade). Their experience ranged from 8 days to 18 years (median = 4 years). Additionally all patients recruited within the National Health Service study were controlled to present with lower back pain, a condition in which the management and treatment options were well evidenced and described within published national guidelines [34]. National Health Service initial consultation times were 45 min and follow-up sessions took 30 min. Despite the significant differences between the physiotherapists’ and our sample’s O12 scores, both study’s scores remain below the accepted 60% threshold score. Sub-threshold O12 scores have also been reported in a study of thirteen Flemish physical therapists who attained a score of 5.2%, which was measured over 237 patient encounters [9].

Lack of practitioner engagement with SDM appears to be common-place in other areas of medicine too. One systematic review of 29 studies using the O12 instrument across a variety of medical professions, calculated an overall mean O12 score of 23% that ranged between 3 and 68% [29]. Couët et al. also noted that those studies which included an intervention designed to improve qualified clinician’s SDM behaviours did all show positive improvements in post-intervention O12 scores; ‘interventions’ included the introduction of decision aids as well as training. Interestingly, one of the reviewed studies using a sample of medical students from different year groups similar to our study, also found no differences in O12 scores between the year groups [35].

### Osteopathic significance of SDM behaviours

SDM appears more effective in situations of repetitive or long term care for chronic problems, creating an open therapeutic relationship that allows treatment decisions to be revisited relatively easily [36]. SDM may also facilitate discussion of potentially serious risks associated with a particular treatment, or where evidence underpinning a particular approach may be lacking [35]. Within osteopathy, many patients present with long-term multi-morbidity or conditions requiring ongoing management [37]. Additionally the technical approaches used by osteopaths do carry rare risks of serious adverse events [38]. The majority of osteopathic post-treatment events, however, can be classified as ‘mild’ or ‘moderate’ [38]. Within one OEI’s teaching clinics, at least 80% of patients do report one or more adverse events in the week following treatment [39].

### Limitations and strengths

There are several issues that may have impacted on this study: The Hawthorne or observer effect is known to change clinical behaviours although the magnitude of its influence is heterogeneous [40]. This effect has also been demonstrated when placing voice recorders in medical doctor's consultations; clinical behaviour significantly improved as evidenced by a reduction in antibiotic prescriptions [41]. In our study, as part of the consent process, all participating students, patients and tutors would be aware that they were being recorded. In addition, throughout the appointment, the two digital voice recorders were in view of the student, patient and tutor. Conceivably, as all parties were aware that they were being recorded, it would have impacted on communication and the quality of verbal communication between parties. It is logical that at the very least, the awareness of being recorded would have had little or no impact on communication between the parties, but there is a real threat that inter-party communication would have become more considered. If the SDM behaviours we did identify resulted from a Hawthorne effect, the implication is that the true number of SDM behaviours would be lower or perhaps absent all together in this cohort. The presence and role of the teaching clinic tutor may also have impacted on all dyad interactions in this study. In this particular Osteopathic Educational Institute, clinical students are obliged to discuss their findings, treatment plan and clinical reasoning with the supervising tutor. Depending on when the tutor entered the room, this could have an impact on the natural flow of communication between the student and patient, resulting in altered SDM behaviours. Future studies on student SDM may wish to capture and incorporate tutor influence on this, indeed capturing data on tutor SDM behaviours would be important to ascertain if appropriate modelling of these behaviours is occurring in osteopathic teaching clinics.

The O12 instrument itself is also acknowledged to have limitation: the frequency of clinician SDM behaviours within a patient encounter is not captured and neither is a patient’s perception of SDM [30, 35]. Additionally, should a patient instigate the SDM process (e.g. by stating a particular fear or by requesting a certain type of treatment), the O12 instrument will not award points for this because the interaction was not initiated by the practitioner; hypothetically, therefore, practitioners who do actively create a relationship that encourages patient autonomy and questioning, may paradoxically end up with relatively low O12 score as most questions would be patient initiated. We chose to use the O12 so that we could directly compare our results with previously published work. Although methodological issues have been noted that may impact on the validity of the O12, its reliability is acknowledged to be sufficient for between group comparisons [42]. The O12 has also been modified and revised and now uses five items (known as the OPTION (5)). Early comparison with the O12 shows ‘excellent’ inter-rater reliability, high correlation (r = 0.71) plus better differentiation of patient involvement [43].

### SDM and osteopathic education

Unsurprisingly, medical clinicians who were specifically trained in the use of SDM achieved higher O12 scores than those who did not receive training [29]. Even brief training allows GPs to successfully implement SDM into practice, leading to improved practitioner and patient satisfaction [3]. Within this OEI, at the time this study was carried out, SDM behaviours were not explicitly included within the taught curriculum and in view of the O12 score obtained by this group of undergraduate clinical students, we suggest that it is vital SDM training is embedded within the pre- and clinical training for undergraduate osteopathic students as well as clinical and technique staff.

Integrating SDM into the school’s teaching requires a multi-faceted approach over the years and theoretical knowledge of SDM could be embedded into pre-clinical curriculum with opportunities for students to practise the behaviours and verbal skills created. This may ideally be positioned within case history and clinical examination classes and SDM behaviours may also be embedded in practical classes and teaching clinic. Including prompts on case history sheets may remind students to be aware of: delivering appropriate information; managing patient expectations and exploring patient preference. Osteopathic Educational Institutes may wish to consider how to support their teaching staff in becoming role models for SDM and support student engagement by leading through example. Provision of SDM training to all existing technical and clinical teaching staff could become part of the Osteopathic Educational Institutes’ ongoing training programme. Integrating SDM training into newly recruited staff induction training would also be beneficial. Without explicit SDM training at either undergraduate or postgraduate level, SDM will probably remain poorly practised. There is also an opportunity for Osteopathic Educational Institutes to design and deliver post-graduate SDM training that would fulfil osteopaths’ Continuing Professional Development training requirements. It is also worth noting that the upcoming revised Continuing Professional Development requirements for United Kingdom osteopaths now has a specific  requirement to update knowledge on 'effective communication' and 'shared decision-making' [44].

Finally, the development and provision of decision support tools to assist patients in reflecting on their feelings about certain types of treatment is essential. The planning and design of these for a complex condition such as low back pain will be challenging [45], the first for example for chronic low back pain has recently been published [46]. Ideally these tools will equally support both practitioner and patient in the SDM journey.

### Areas of future research

As far as we know, this is the first time the O12 has been used within an undergraduate osteopathic educational setting. In view of the known benefits of SDM, combined with the legal requirement for osteopaths to use SDM, we think that it is important for the United Kingdom Osteopathic Educational Institutes to benchmark SDM behaviours within their clinical teaching and student populations using either the O12 or more recently developed 5 item OPTION instrument [47]. Additionally, using an OPTION instrument to capture data on SDM behaviours in practising osteopaths within the UK and internationally would allow identification of a potential training opportunity that would enhance patient management.

## Conclusion

Despite SDM being embedded within United Kingdom statutory osteopathic practice standards, students at one Osteopathic Educational Institute did not implement SDM to an acceptable level within their practice. This suggests that the students were using more traditional and paternalistic approaches to decision making and patient care. A similar lack of SDM engagement also appears in other manual therapy and medical professions. Since SDM has been shown to be more important in longer-term treatment and where treatment carries a risk of serious side effects, we think it is imperative that Osteopathic Educational Institutes audit clinical educator and student SDM behaviours and prioritise remediation of any deficits identified in this area. We think Osteopathic Educational Institutes may also wish to explore development and use of decision aids within their teaching clinics.

## Data Availability

The datasets generated and/or analysed during the current study are not publicly available due to the fact they are audio recorded interviews. Anonymised transcripts are available from the corresponding author on reasonable request.

## Abbreviations

O12- OPTION-12: Observing Patient Involvement instrument (12 items)
SDM: Shared Decision Making
